# Methods for time series analysis of RNA-seq data with application to human Th17 cell differentiation

**DOI:** 10.1093/bioinformatics/btu274

**Published:** 2014-06-11

**Authors:** Tarmo Äijö, Vincent Butty, Zhi Chen, Verna Salo, Subhash Tripathi, Christopher B. Burge, Riitta Lahesmaa, Harri Lähdesmäki

**Affiliations:** ^1^Department of Information and Computer Science, Aalto University, FI-00076 Aalto, Finland, ^2^Department of Biology, Massachusetts Institute of Technology, Cambridge, MA 02139, USA and ^3^Turku Centre for Biotechnology, University of Turku and Åbo Akademi University, FI-20520 Turku, Finland

## Abstract

**Motivation:** Gene expression profiling using RNA-seq is a powerful technique for screening RNA species’ landscapes and their dynamics in an unbiased way. While several advanced methods exist for differential expression analysis of RNA-seq data, proper tools to anal.yze RNA-seq time-course have not been proposed.

**Results:** In this study, we use RNA-seq to measure gene expression during the early human T helper 17 (Th17) cell differentiation and T**-**cell activation (Th0). To quantify Th17**-**specific gene expression dynamics, we present a novel statistical methodology, DyNB, for analyzing time-course RNA-seq data. We use non-parametric Gaussian processes to model temporal correlation in gene expression and combine that with negative binomial likelihood for the count data. To account for experiment**-**specific biases in gene expression dynamics, such as differences in cell differentiation efficiencies, we propose a method to rescale the dynamics between replicated measurements. We develop an MCMC sampling method to make inference of differential expression dynamics between conditions. DyNB identifies several known and novel genes involved in Th17 differentiation. Analysis of differentiation efficiencies revealed consistent patterns in gene expression dynamics between different cultures. We use qRT-PCR to validate differential expression and differentiation efficiencies for selected genes. Comparison of the results with those obtained via traditional timepoint**-**wise analysis shows that time-course analysis together with time rescaling between cultures identifies differentially expressed genes which would not otherwise be detected.

**Availability:** An implementation of the proposed computational methods will be available at http://research.ics.aalto.fi/csb/software/

**Contact:** tarmo.aijo@aalto.fi or harri.lahdesmaki@aalto.fi

**Supplementary information:** Supplementary data are available at *Bioinformatics* online.

## 1 INTRODUCTION

A RNA-seq experiment provides a snapshot of RNA content within a cell population. The observed data is in a form of millions of short nucleotide sequences, which can be used to construct a *de novo* transcriptome or aligned against known reference genome and transcriptome. To quantify expressions of known genes, a common approach is to count the reads which are aligned to different genes. The discrete nature of count data led researchers to model the sequencing data using Poisson distribution (see e.g. Marioni *et al.* 2008). Recently, it has been shown that the Poisson distribution is insufficient for modeling sequencing data because it tends to underestimate the variance for highly expressed genes. An extension of the Poisson distribution, the negative binomial distribution, has gained popularity in modeling gene expression data from RNA-seq (or other sequencing-based count data) because it can account for this over-dispersion. Two commonly used approaches which use the negative binomial distribution to detect differential expression are DESeq (Anders and Huber, 2010) and edgeR (Robinson *et al.*, 2010). Another method called baySeq uses an empirical Bayesian method to estimate the posterior probabilities that a gene is, or is not, differentially expressed (Hardcastle and Kelly, 2010).

Profiling gene expression over time provides information about the dynamical behavior of the genes. Storey *et al.* (2005) presented a method that can analyze time series microarray data in order to assess the differential expression from whole time series as opposed to the traditional methods, which analyze timepoints independently. More recently, Stegle *et al.* (2010) presented a methodology that uses Gaussian processes (GPs) to model gene expression over time and to identify the time intervals when each gene is differentially expressed. We have further extended the GP approach to quantify condition-specific differential expression among multiple time-course experiments (Äijö *et al.*, 2012). These methodologies are not optimal for analyzing count data due to the different statistical characteristics and, to our knowledge, next-maSigPro (Conesa and Nueda, 2013) is the only methodology capable of taking into account the temporal dimension of RNA-seq time series. In addition, by taking into account temporal correlation makes it possible to carry out more detailed analysis of the observed dynamics, e.g. to quantify similarities and differences between the observed kinetics. To that end, GPs have been used for modeling temporally or spatially varying likelihood parameters in other fields, e.g. to model the rate parameter of the Poisson distribution temporally and the stochastic process that is produced is called as the Gaussian Cox process (Adams *et al.*, 2009). Similar approaches have also been popular in geostatistics (Diggle *et al.*, 1998).

Since the discovery of an interleukin 17 producing T-cell subset, this T helper 17 (Th17) cell lineage has been a focus of great research interest (Dong, 2008; Park *et al.*, 2005). Th17 cells have been shown to play an important role in autoimmune diseases and inflammation. Recent studies have identified transcription factor genes *Rorc* and *Stat3* as the key regulators of the early Th17 differentiation in murine (see a review in Ivanov *et al.*, 2007). Naïve human T cells are activated through the T-cell receptor (TCR) by αCD3 and αCD28 and Th17 cells are polarized from the activated T cells by exposing the cells to TGF-β, IL-1β and IL-23. The goal of gene expression profiling in the early phase of Th17 differentiation is to gain insight into the process of differentiation by unraveling dependencies between key factors and to understand how the differentiation signal propagates through various pathways and gene regulatory networks. This knowledge could potentially prove useful in identifying biomarkers for immune-related diseases and in design of therapeutic interventions.

We present a methodology, DyNB that is built on the negative binomial likelihood and GPs. Non-parametric GP regression is used to model gene expression over time and the model inference is carried using the Bayesian reasoning. We demonstrate the applicability of DyNB by analyzing RNA-seq time-series datasets. We also show how DyNB can be used to study relative differentiation efficiencies between biological samples. The differentially expressed genes detected by DyNB as well as estimated differences in differentiation efficiencies for selected genes are validated using qRT-PCR.

## 2 METHODS

### 2.1 GPs

The GP prior for functions is a collection of random variables such that distribution for any finite subset (index set) *X* is defined as

(1) F|X,θ∼N(m,K),

where **F** represents the process at X,θ is the set of hyperparameters, **m** is the mean of the process, and *K* is the covariance matrix. In our application, the index set *X* of the random variables is time. We define the covariances between pairs of random variables as follows

(2) $$\text{Cov}\left(F(t_{p}),F(t_{q})\right)=k(t_{p},t_{q})=\theta_{1}\exp\left(-\frac{1}{2\theta_{2}}{\left|t_{p}-t_{q}\right|}^{2}\right),$$

where k(·,·) is the squared exponential covariance function and $\theta ={\left(\theta_{1},\theta_{2}\right)}^{\text{T}}$. The ${\left(i,j\right)}^{\text{th}}$ element in the matrix *K* is given by $k(t_{i},t_{j})$.

### 2.2 A time-varying negative binomial distribution

Read count data are commonly modeled using the negative binomial distribution (Anders and Huber, 2010; Robinson *et al.*, 2010)

(3) Y∼NB(r,p),

where *r* is a predefined number of failures and the probability of success is *p*. We will parameterize the negative binomial distribution with mean μ=E{Y}=pr/(1−p) and variance $\sigma^{2}=\text{Var}\{Y\}=pr/{\left(1-p\right)}^{2}$. Thus, we solve *p* as a function of μ and $\sigma^{2}$ as

(4) $$p=\frac{\sigma^{2}-\mu }{\sigma^{2}}$$

and similarly *r*

(5) $$r=\frac{\mu^{2}}{\sigma^{2}-\mu },$$

hence we can write $Y\;∼\;\text{NB}\left(\mu ,\sigma^{2}\right)$. We assume to have *M* replicates (j=1,…,M) in *N* timepoints (i=1,…,N), i.e., $Y_{j}(t_{i})\;∼\;\text{NB}\left(\mu (t_{i}),\sigma {\left(t_{i}\right)}^{2}\right)$. Observed read count data $y_{j}(t_{i})(j=1,\ldots ,M,i=1,\ldots ,N)$ is collectively denoted as **y**. We omit the index of a gene for notational simplicity.

Let us write the mean of the negative binomial distribution as a function of a random process **F**, i.e. $Y\;∼\;\text{NB}\left(g_{1}(f),\sigma^{2}\right)$, where **f** is a realization of a GP. In the case of a GP, we define $g_{1}(f(t_{i}))=f(t_{i})$, where $f(t_{i})$ is a value of the random process at the *i*-th timepoint. Then we can write the likelihood of the data as follows (see Supplementary Equations S2 and S3)
(6)
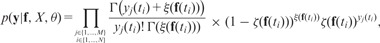

where

(7) $$\xi (f(t_{i}))=\frac{g_{1}^{2}\left(f(t_{i})\right)}{\sigma {\left(t_{i}\right)}^{2}-g_{1}\left(f(t_{i})\right)}$$

and

(8) $$\zeta (f(t_{i}))=\frac{\sigma {\left(t_{i}\right)}^{2}-g_{1}\left(f(t_{i})\right)}{\sigma {\left(t_{i}\right)}^{2}}.$$

### 2.3 A time-varying negative binomial distribution with time scaling

We also consider a situation where we possess a priori knowledge that the biological replicates are differentiating in different time scales. In this study, we assume that the different time scales between biological replicates can be modeled as $t_{j}=t/k_{j}$. The different time scales are taken into account via the GP realizations $f(t_{j}),j=1,\ldots ,M$
(9)
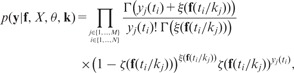

where $k=(k_{1},\ldots ,k_{M})$ are the replicate-specific time-scaling factors. Often one wants to analyze time scaling with respect to one of the replicates, e.g., *i*-th replicate, which can be achieved by constraining $k_{i}=1$. This also makes the model identifiable.

The statistical dependencies of the variables in our model are depicted in Supplementary Figure S1 using the plate notation.

### 2.4 Variance estimation and normalization

The variance for the negative binomial distribution is estimated using the approach described in Anders and Huber (2010), i.e. we model the variance as a function of the read count using a smooth function. The idea behind the variance estimation is that genes expressed in a similar level have a similar variance and sharing information between genes improves variance estimation (Anders and Huber, 2010). In other words, $\sigma {\left(t_{i}\right)}^{2}$ in Equations (7) and (8) are obtained from a polynomial of degree 2 giving a robust variance estimate as a function of the read count. The second order polynomial is fitted to the observed read counts and variances across timepoints and genes.

To account for different sequence depths of the samples (over all the replicates and timepoints), we make the read counts between different RNA-seq runs comparable by scaling factors, which are estimated using the procedure presented in Anders and Huber (2010). Instead of scaling the discrete read counts, the scaling is performed on the GPs samples **f**.

### 2.5 Bayesian inference for transcriptome dynamics

By using the likelihood in Equation (9), we can write the marginal likelihood

(10) p(y|X,θ,k)=∫p(y|f,X,θ,k)p(f|X,θ,k)df,

where we have marginalized over the possible realizations of **F**. In this case, the integral in Equation (10) is not analytically tractable and we resort to Markov chain Monte Carlo (MCMC) methods. A common practice is to marginalize over all the parameters, which in our case include **f**, **k** and θ

(11) $$p(y|X)=\int \overset{\text{NB distribution}}{\overset{︷}{p\left(y|f,X\right)}}\left(\underset{=p\left(f|X\right)}{\underset{︸}{\int \left(\int \overset{\text{Gaussian distribution}}{\overset{︷}{p\left(f|X,\theta ,k\right)}}\overset{\text{prior}}{\overset{︷}{p\left(\theta \right)}}\text{d}\theta \right)\overset{\text{prior}}{\overset{︷}{p(k)}}\text{d}k}}\right)\text{d}f.$$

For the integration in Equation (11), we construct a Metropolis–Hasting algorithm presented in Algorithm 1.

**Algorithm 1 A Metropolis–Hastings algorithm for posterior sampling of parameters θ,k and f.**

**Require:** y,X

**Initialize:** $\theta^{\left(0\right)},k^{\left(0\right)},f^{\left(0\right)}$

 **for** *i* = 0 to *N* – 1 **do**

  **Sample:** $u\;∼\;U_{\left[0,1\right]}$

  **Sample:** $\theta^{*}\;∼\;q_{\theta }(\theta^{*}|\theta^{\left(i\right)})$

  **Sample:** $k^{*}\;∼\;q_{k}(k^{*}|k^{\left(i\right)})$

  **Sample:** $f^{*}\;∼\;q_{f}(f^{*}|f^{\left(i\right)},X,\theta^{*})$

  **if** $u<\text{min}\{1,\frac{p_{\theta }(\theta^{*})p_{k}(k^{*})p_{f}(f^{*})p(y|f^{*},k^{*},X)}{p_{\theta }(\theta^{\left(i\right)})p_{k}(k^{\left(i\right)})p_{f}(f^{\left(i\right)})p(y|f^{\left(i\right)},k^{\left(i\right)},X)}\times \frac{q_{\theta }(\theta^{\left(i\right)}|\theta^{*})q_{k}(k^{\left(i\right)}|k^{*})q_{f}(f^{\left(i\right)}|f^{*},X,\theta^{*})}{q_{\theta }(\theta^{*}|\theta^{\left(i\right)})q_{k}(k^{*}|k^{\left(i\right)})q_{f}(f^{*}|f^{\left(i\right)},X,\theta^{\left(i\right)})}\}$   **then**

   $\theta^{\left(i+1\right)}\leftarrow \theta^{*},k^{\left(i+1\right)}\leftarrow k^{*},f^{\left(i+1\right)}\leftarrow f^{*}$

**  else**

   $\theta^{\left(i+1\right)}\leftarrow \theta^{\left(i\right)},k^{\left(i+1\right)}\leftarrow k^{\left(i\right)},f^{\left(i+1\right)}\leftarrow f^{\left(i\right)}$

** end** if

**end for**

We assign the uniform prior distribution for the hyperparameter θ_2_, i.e. $p_{\theta }(\theta )=U[0.5,1]$, to favor smooth GP realizations and a symmetric prior $p_{k}(\cdot )$ for time-scaling factors $k_{j},j=1,\ldots ,M$, which is centered around identity scaling (Fig. 5A). The parameter θ_1_ is set empirically to account for large differences in gene expression counts **y** between low- and high-expressed genes (from 1 to approx. $5\times 10^{5}$ in our case). Thus, θ_1_ is fixed to a gene-specific and data-dependent value 10 Stdev{y}. The GP prior per gene ($p_{f}$) is defined by the mean vector and covariance matrix, which is parameterized by the parameters σ_1_ and σ_2_ (which have a similar role as θ_1_ and θ_2_). Again, in defining the mean **m** and σ_1_, we should take into account the large range of different read count magnitudes; thus they are defined separately for each of the genes. The mean vector is defined as $m=\frac{\text{Max}\{y\}+\text{Min}\{y\}}{2}1$ and $\sigma_{1}=500\frac{\text{Max}\{y\}+\text{Min}\{y\}}{2}$ and $\sigma_{2}=0.75$.

In our implementation, we use a truncated normal distribution as the proposal distribution $q_{\theta }$, where the last accepted sample $\theta_{2}^{\left(i\right)}$ is the mean and the variance and the boundaries are predefined, i.e. $N_{\left[0.5,1\right]}(\theta_{2}^{\left(i\right)},0.01^{2})$. Our choice of the proposal distribution for the time-scaling factors **k** is an uniform distribution, where the probabilities for the three allowed transitions, i.e. +4 h, −4 h and 0 h, are 1/3. For the proposal distribution $q_{f}$ we use the GP prior whose mean is the last accepted sample $f^{\left(i\right)}$ and the covariance matrix *K* is defined by the inputs *X* and the hyperparameters $\theta^{*}$.

Using the accepted samples $f^{\left(i\right)}$ we estimate the posterior mean and (co)variance of the distribution using the standard sample estimators. The marginal likelihood is estimated using the harmonic mean of the likelihoods of the samples from the posterior distribution as presented in Newton and Raftery (1994), where the idea is to use the parameter posterior as the importance sampling function

(12) $$\begin{array}{l} p(y|X)\approx {\left(\frac{1}{m}\sum_{i=1}^{m}p{\left(y|X,f^{\left(i\right)},\theta^{\left(i\right)},k^{\left(i\right)}\right)}^{-1}\right)}^{-1},\\ \text{where }f^{\left(i\right)},\theta^{\left(i\right)},k^{\left(i\right)}\;∼\;p(f,\theta ,k|y). \end{array}$$

Another variable whose posterior distribution we are interested in is **k** whose posterior we also get directly from the MCMC chain. Moreover, the estimated marginal likelihoods are used for model selection purposes as we will see in next section. The convergence of the chains was assessed using the potential scale reduction factors as described in Gelman and Rubin (1992), and the results confirming the convergence are depicted in Supplementary Figure S2.

### 2.6 Quantification of differential dynamics

In this study we want to answer the question whether a gene is differentially expressed between different conditions, namely Th0 and Th17 lineages, and we assume to have replicated time series measurements from these two lineages. From now on we consider only two conditions but the same methodology can be easily generalized for any number of conditions (Hardcastle and Kelly, 2010; Äijö *et al.*, 2012). The null model, $M_{0}$, denotes that the Th0 and Th17 lineages behave similarly, which we implement by fitting a single DyNB model to Th0 and Th17 measurements. The alternative model, $M_{1}$, denotes that the two lineages behave differently, which we implement by fitting one DyNB model to Th0 and another DyNB model to Th17. Assuming equal prior probabilities for both models, the evidence for the alternative model is quantified by the Bayes factor (BF)

(13) $$\text{BF}=\frac{p(y|X,M_{1})}{p(y|X,M_{0})}.$$

By following the BF interpretation chart described in Jeffreys (1998), a BF ≥10 should be thought as strong evidence for the model $M_{1}$ over the model $M_{0}$. BFs were recently used for model selection purposes in the context of identifying alternative splicing events between biological samples (Katz *et al.*, 2010).

### 2.7 Human-activated T and Th17 cells

**Fig. 1.**
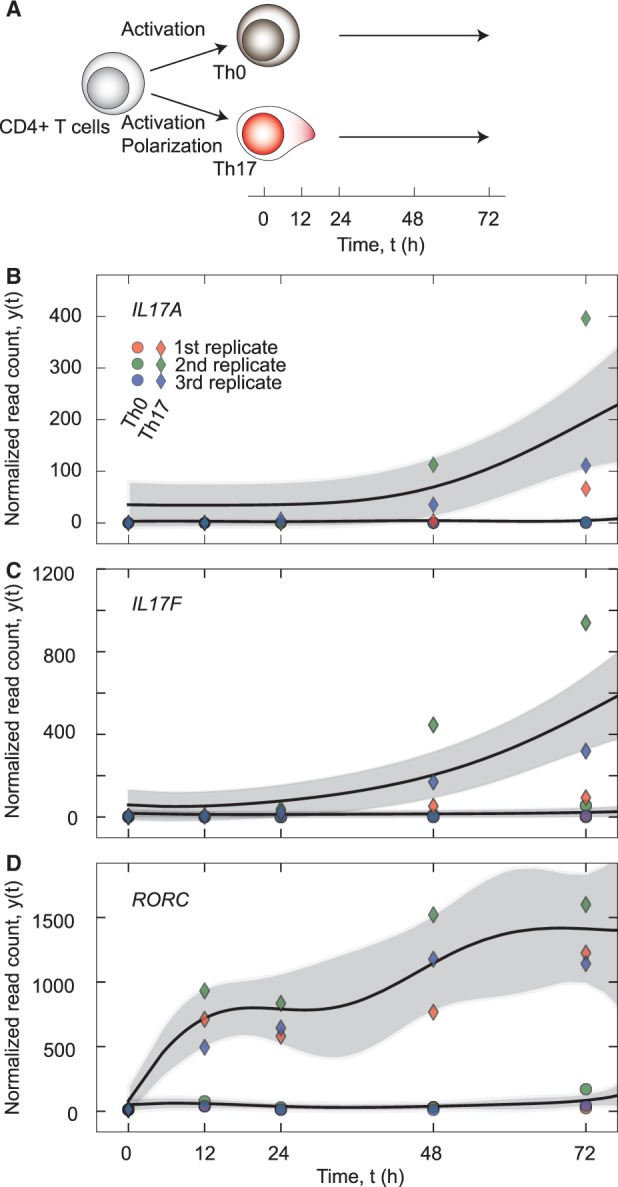
Transcriptome dynamics of Th17 marker genes. (**A**) T helper precursor cells isolated from cord blood are activated using plate-bound αCD3 and soluble αCD28 in the presence of αIFN-γ and αIL-4 yielding the cells to follow the Th0 lineage. Th17 commitment is achieved by activation and polarization condition, including IL-6, IL-1β and TGF-β. Cells were harvested at 0, 12, 24, 48, and 72 h. From the harvested cells the RNA was extracted and used for preparation an RNA-seq library. (**B**) The estimated smooth representation of *IL17A* dynamics without time scaling. The read counts are on the *y*-axis. Circles and diamonds mark the measurements from Th0 and Th17 cells, respectively, and the replicates are distinguish with different colors. The solid curves are the posterior means of the specific Th0 and Th17 models ($M_{1}$) with corresponding 95% CIs (shaded areas around means). (**C** and **D**) Same as (B), but the depicted results are for the *IL17F* and *RORC* genes

CD4^+^ T cells were isolated from the umbilical cord blood collected from healthy neonates born in Turku University Hospital; Hospital District of Southwest Finland with approval from the Finnish Ethics Committee. CD4^+^ T cells were isolated from umbilical cord blood samples using Ficoll-Paque and anti-CD4 magnetic beads. For activating CD4^+^ T cells and inducing polarization of Th17 phenotype the cells were activated and stimulated as indicated in Figure 1A and as previously described (Tuomela *et al.*, 2012). The polarization was confirmed as described by Tuomela *et al.* (2012). Strand-specific RNA-seq libraries were prepared from 2–5 µg of total RNA (Parkhomchuk *et al.*, 2009), bar-coded and multiplexed (3 to 4 samples per lane) and 40-nt paired-end reads were obtained on an Illumina HiSeq2000. The gene expressions were profiled from Th0 and Th17 cells at the five timepoints, 0, 12, 24, 48 and 72 h with three biological replicates. The Ensembl gene models were used in the gene expression estimation.

## 3 RESULTS

### 3.1 Temporal modeling of RNA-seq data

Using the model described in Section 2, our first goal is to estimate a smooth representation of gene expression dynamics based on the measured read counts. Smoothness of expression dynamics is enforced by the GP prior, and agreement of expression dynamics with the read count data is quantified using the negative binomial likelihood. To avoid overfitting, the inference is done using the Bayesian analysis, and thus the final model fitting estimate is obtained by integrating over parameters using an MCMC sampling technique.

Applying the aforementioned methodology without the time-scaling option to RNA-seq data, we estimated the smooth representations of the underlying gene expression in Th0 and Th17 lineages. The posterior means (solid curves) of the specific Th0 and Th17 models ($M_{1}$) together with corresponding 95% CIs (shaded areas around means) for *IL17A*, *IL17F* and *RORC* are depicted in Figures 1B–D.

For example, the cytokine IL17A is known to be highly expressed in Th17 cells and its expression is commonly used to assess the Th17 polarization efficiency (Brucklacher-Waldert *et al.*, 2009). The strong induction of *IL17A* and *IL17F* in the Th17 differentiation is apparent by the data. Based on visual assessment, however, the induction of *IL17A* and *IL17F* behaves differently among the replicates.

### 3.2 Modeling of variable differentiation efficiency

**Fig. 2.**
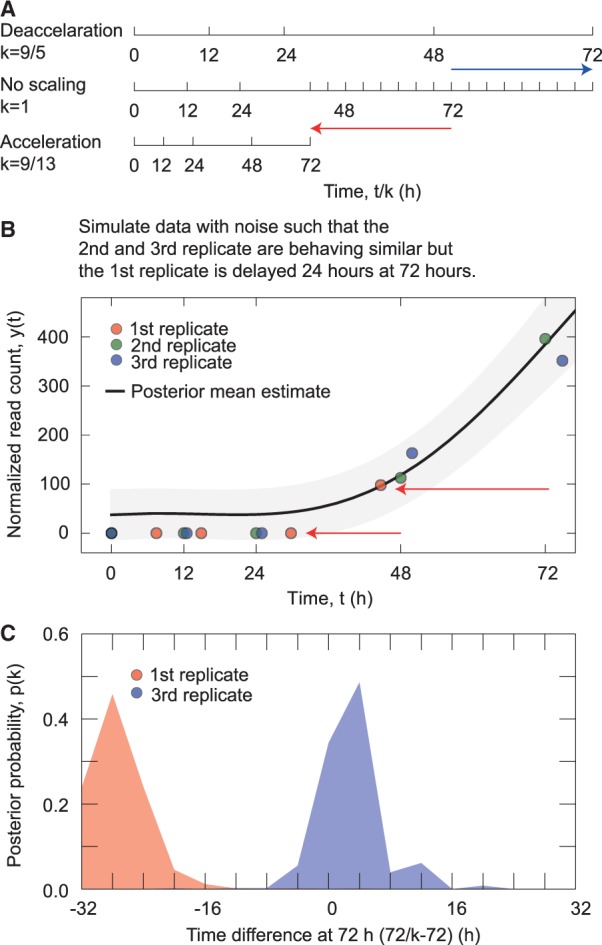
Modeling differentiation dynamics**.** (**A**) An illustration showing the effects of the time scaling. The axis in the center panel shows the unscaled time axis. The axes in the top and bottom panels show the maximum allowed deceleration (−32 h at 72 h) and acceleration (32 h at 72 h) relative to the unscaled case, respectively. (**B**) The estimated smooth representation of the simulated data with the time scaling. The first replicate is a delayed version of the second and third replicates. The red arrows illustrate how much the measurements are effectively moved due to the time scaling. (**C**) The posterior distribution of the time differences at timepoint 72 h

To study variable differentiation efficiencies in IL17 genes in an unbiased manner, we repeated the analysis but now taking into account the possibility of different time scales between the replicates. The model with time scaling allows the samples to be decelerated/accelerated relatively to each other, so that their scaled behavior is similar. We fixed the time scale of the second sample and allowed the other two samples to be accelerated or decelerated independently of each other using the transformation $t/k_{j}$. Another choice could have been a time shift, $t+s_{j}$, which moves linearly the whole time series together with the start point. Because in our case the cells are activated and polarized exactly at the same time, we wished to keep the start point fixed across the samples. The transformation is illustrated in Figure 2A, where the axis in the center corresponds to the case without time scaling and the top and bottom axes correspond the cases of 32 and −32 h time differences at 72 h due to the time scaling (corresponding to *k* = 5/9 and *k* = 13/9), respectively. We constrained the effects of scaling to be discrete, i.e. from −32 to +32 h at the end of the time series (72 h) in 4 h steps. To demonstrate the methods applicability for estimating differentiation efficiencies, we carried out a simulation study. Using *IL17A* as a template profile, we generated two time series (2nd and 3rd replicate) with a similar behavior and third one (the first replicate) which is a delayed version of the two, i.e. the timepoint 72 h corresponds to 48 h. The method correctly inferred that the first replicate is delayed compared with the other two replicates as depicted in Figure 2B. Finally, the estimated posterior distributions of time differences depicted in Figure 2C demonstrated the method’s accuracy in estimating differences in differentiation efficiency.

**Fig. 3.**
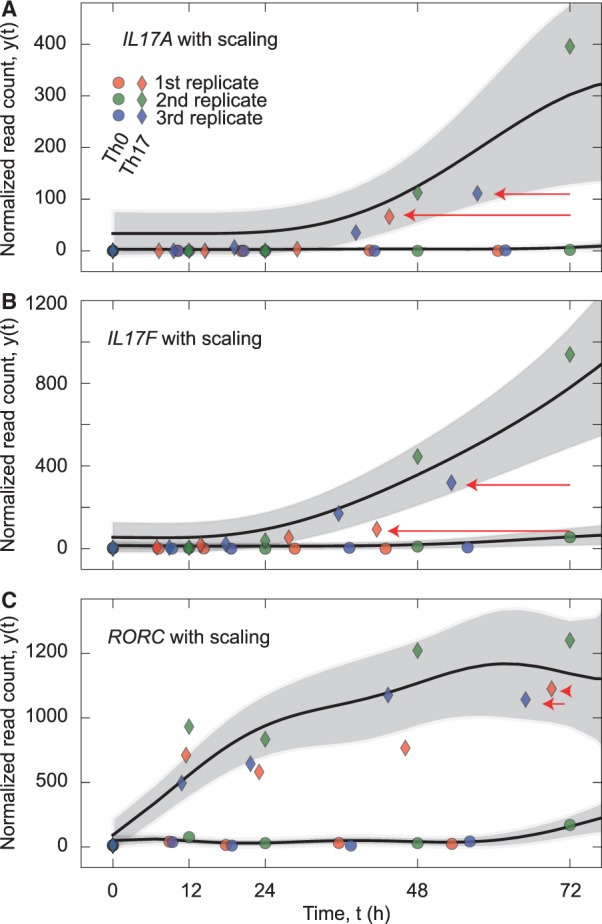
Perturbated differentiation dynamics. (**A**) The estimated smooth representation of *IL17A* dynamics with the time scaling. The red arrows illustrate how much the measurements are effectively moved due to the time scaling. (**B** and **C**) Same as (A), but the depicted results are for the *IL17F* and *RORC* genes

**Fig. 4.**
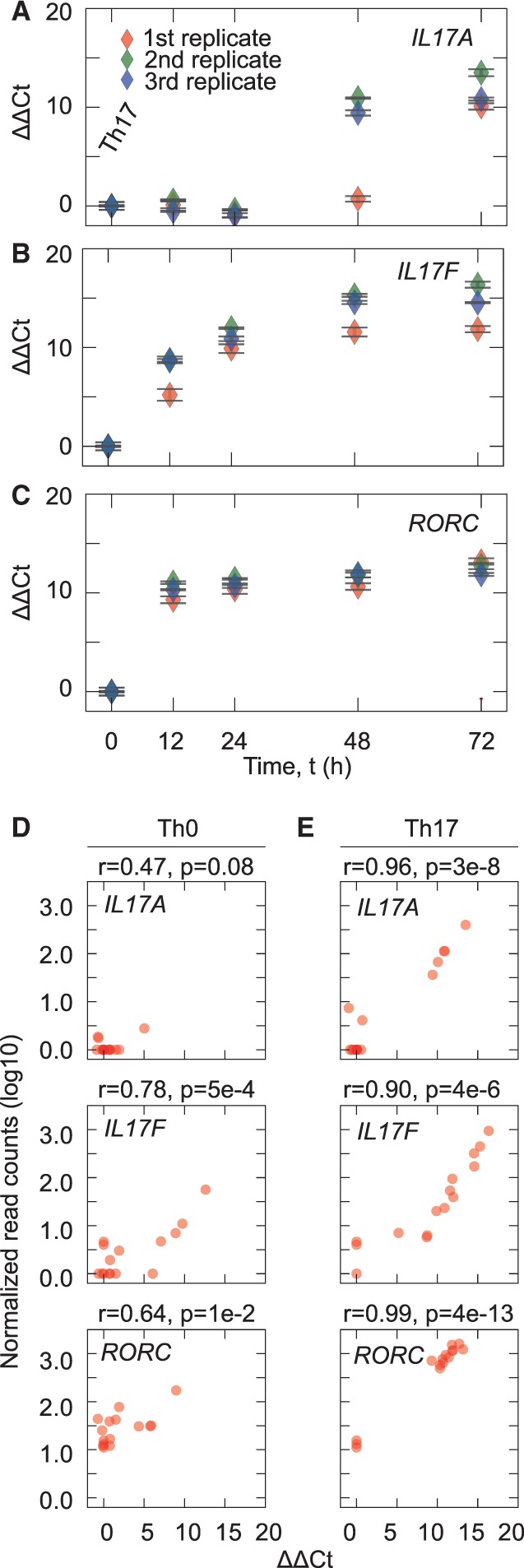
Validation of marker gene expression**.** (**A**) qRT-PCR time-series measurements of *IL17A* mRNA levels in the same samples where RNA-seq was performed. The error bars are depicting the SDs. The colors distinguish the different samples. (**B** and **C**) Same as (A) but for *IL17F* and *RORC*, respectively. (**D**) The scatter plots illustrating the replicate-specific correspondence between the qRT-PCR and RNA-seq gene expression estimates of the *IL17A* (top panel), *IL17F* (middle panel) and *RORC* genes (bottom panel) over time in Th0 cells. The correlation is quantified using the Pearson correlation coefficient (*r*). (**E**) Same as in (D) but for Th17 cells

The results with time scaling for the marker genes *IL17A*, *IL17F* and *RORC* are depicted in Figures 3B–D, respectively. The effect of time scaling is visualized by transforming the measurements based on the time-scaling parameter posterior mean: e.g. *IL17A* is delayed over 24 h at 72 h in the first Th17 sample. As expected, uncertainty of the estimates, especially at the end of time series, increases due to the time scaling. For the marker genes *IL17A* and *IL17F*, however, we notice that the time scaling is able to improve the model fit. To validate our observation of different time scaling, we performed a kinetic assay of *IL17A*, *IL17F* and *RORC* mRNA levels throughout the early Th17 differentiation using qRT-PCR in the same biological samples as the RNA-seq (Fig. 4; Supplementary Table S1). Note that because time scaling (i.e. differentiation efficiency) is a replicate-specific random effect we need to use the same samples for qRT-PCR validation. These confirmed our conclusions: expression of *IL17F* and *IL17A* was delayed in the first and third series, while expression of *RORC* behaved similarly across the samples.

**Fig. 5.**
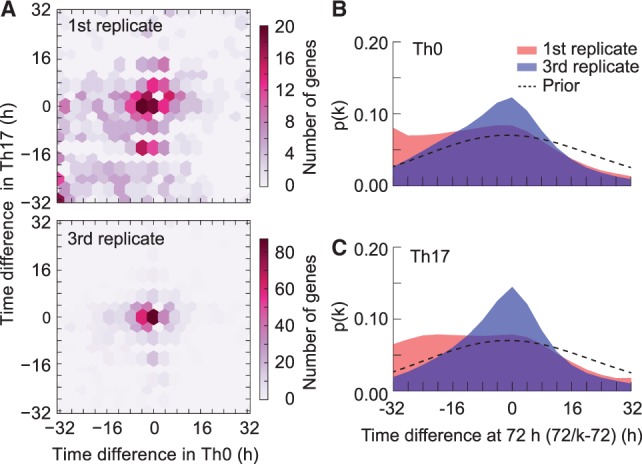
The replicate-specific differentiation efficiencies**.** (**A**) Density plots representing the distribution of estimated time differences in gene level in the Th0 and Th17 lineages. A gene is on diagonal if the estimated time differences in the Th0 and Th17 cells are the same. The results for the first and third replicate are depicted in top panel and bottom panel, respectively. (**B**) Presence of time scaling in Th0 lineage among the 698 differentially expressed genes. The dashed line represents the prior distribution of the amount of time scaling at 72 h. The red area shows the posterior distribution of the time scaling for the first replicate and the purple shows the posterior distribution for the third replicate. (**C**) Same as (B) but here the focus is on Th17 lineage. The focus is on the differentially expressed genes in (B and C)

Next we wanted to confirm the presence of different time scaling by studying the posterior distribution of the time-scaling genome wide by repeating the analysis for all expressed genes (i.e. at least one read in Th0 and Th17 samples). To detect differentially expressed genes between the Th0 and Th17 lineages, we used the following criteria: (i) BF > 10, i.e. strong evidence for $M_{1}$ over $M_{0}$, and (ii) fold-change > 2 in at least one timepoint. These criteria gave us 698 differentially expressed genes. Then we studied how presence and absence of estimated time-scaling parameters differ between the Th0 and Th17 lineages for each of the differentially expressed genes. The results are depicted as 2D histograms in Figure 5A where the first (top panel) and third replicate (bottom panel) are analyzed separately. In both replicates, there are many genes with no time scaling effect, and thus they behave similarly to the second replicate. In the first replicate, the probability mass is partly distributed to the left lower quadrant, which corresponds to cases where a gene is decelerated in both lineages in the first replicate relative to the second replicate. We can conclude that in terms of genome-wide expression dynamics the first and third replicates are different from each other and that the third and second replicates are similar to each other since the mass in Figure 5A (bottom panel) is centered strongly around the point (0, 0).

Figure 5B and C shows the distributions of time differences between the replicates over all the differentially expressed genes for Th0 and Th17 lineages, respectively. Histograms in Figure 5B and C suggests that both the activation (Th0) and differentiation (Th17) are delayed in the first replicate. We did not observe a difference in differentiation efficiencies for all differentially expressed genes but there is clear shift of the probability mass towards deceleration. Whereas, for the third replicate the posterior distribution is centered around the region corresponding to no time scaling. We conclude that the first replicate differs from the other replicates in its differentiation kinetics.

### 3.3 Comparison of temporal and timepoint-wise analysis

**Fig. 6.**
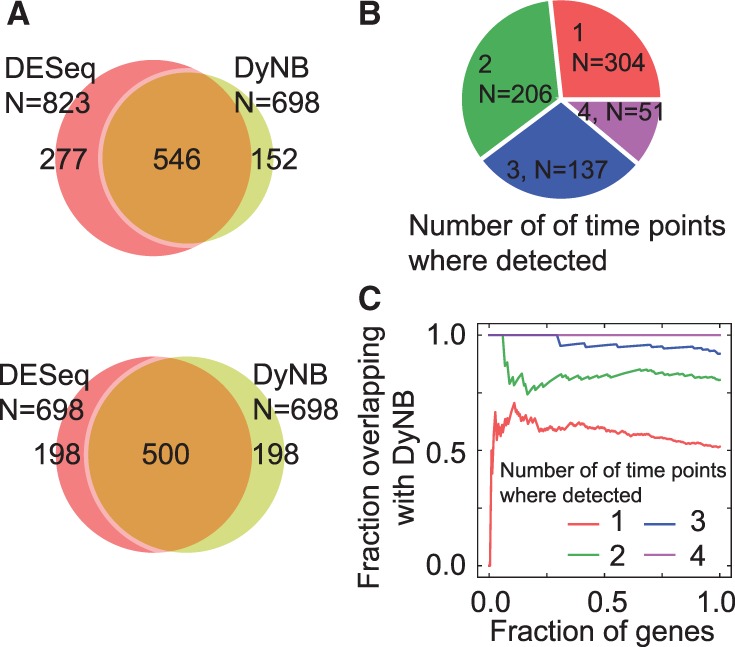
A comparison of the results with DESeq. (**A**) The overlap between the sets of differentially expressed genes identified by DyNB and DESeq (top panel). In the bottom panel we take into account only the top 698 hits from DESeq analysis to make the gene sets equal in size. (**B**) The number of the top 698 DESeq hits that are found to be differentially expressed exactly at one, two, three or four timepoints in the DESeq analysis. (**C**) A quantification of how the genes belonging to the classes presented in (**B**) are found by the presented method using the precision metric. The DESeq hits are taken into account in the order of descending significance (*x*-axis), which are used to evaluate precisions. For example, precision is one when all the considered genes are found in the set given by DyNB

In order to study advantages and disadvantages of our temporal analysis, we carried out a differential expression analysis at the individual timepoints using DESeq tool for comparison purposes. For each timepoint we call a gene differentially expressed if multiple testing corrected (Benjamini–Hochberg method) $p_{\text{adj}}<0.01$ and the absolute value of the $\log_{2}$ fold-change is >1. Combining differentially expressed genes from different timepoints, timepoint-wise analysis gives a total of 823 genes, which is in agreement with the number detected by DyNB. Comparing directly the numbers of genes detected by the frequentist DESeq and our Bayesian DyNB may not be exactly meaningful due to differences in defining the detection thresholds, and simply because timepoint-wise analysis has four times more differential expression tests. Instead, results from the two methods need more careful investigation. Overlap of the differentially expressed genes identified by the two approaches, DyNB and DESeq, are depicted in Figure 6A (top panel). Out of 698 differentially expressed genes identified by DyNB, 546 are also detected by the DESeq. Figure 6A (bottom panel) shows a similar Venn diagram but now using only the top 698 genes from the timepoint-wise analysis (ranked according to the adjusted *P*-values). In this case, 500 genes overlap between temporal and timepoint-wise analysis. The overall agreement between the two methods is demonstrated by the hypergeometric test of gene set overlap (*P* < 1 e−16).

Next we wanted to see how the overlap between temporal and timepoint-wise analysis changes when we consider separately the top 698 genes that are identified by DESeq exactly at one, two, three, or four timepoints. The number of genes belonging to each class is shown in Figure 6B. The agreement between the two methods for different gene classes was quantified using the precision–recall metric as a function of the statistical significance from DyNB analysis (Fig. 6C). As expected, the level of agreement between the presented method and DESeq correlates with the number of timepoints where DESeq identified genes to be differentially expressed. For example, the genes differentially expressed in all four timepoints based on the DESeq analysis are all detected by DyNB as well. We conclude that, on average, both the temporal and timepoint-wise analysis detect largely the same genes, which have a strong differential expression, as expected. However, the overlap is not perfect and different results are reported for genes whose differential expression is weaker or noise level higher and for genes which are affected by variable differentiation efficiency. Additionally, DyNB provides insights into differentiation efficiencies between biological replicates, which is not possible with timepoint-wise or traditional temporal methodologies.

DyNB allows each gene to have its own time scalings between replicates. Thus, we studied the effect of the assumption that all genes would be affected similarly by the differential differentiation efficiency. This was done by introducing informative delay priors (Supplementary Fig. S3A), which closely resembles the posterior distribution of time-scaling parameters obtained from the application of DyNB (Fig. 6C). After applying DyNB with the strong time-scaling prior, we noticed that the distributions of the estimated time differences of the differentially expressed genes (the same criteria as before) resembled the informative prior distributions as depicted in Supplementary Figure S3B, indicating that the time differences can be estimated even without strong regularization. Consequently, we believe that it is more beneficial to apply DyNB without the informative prior distribution because, e.g. in the context of Th17 differentiation only a fraction of genes respond to the differentiation.

We also compared DyNB (with and without the informative delay prior) with the next-maSigPro (Conesa and Nueda, 2013). Interestingly, next-maSigPro (Benjamini-Hochberg-corrected *P*-value <0.01 with the negative binomial model) showed the weakest level of agreement with the other methods as depicted in Supplementary Figure S3C.

**Fig. 7.**
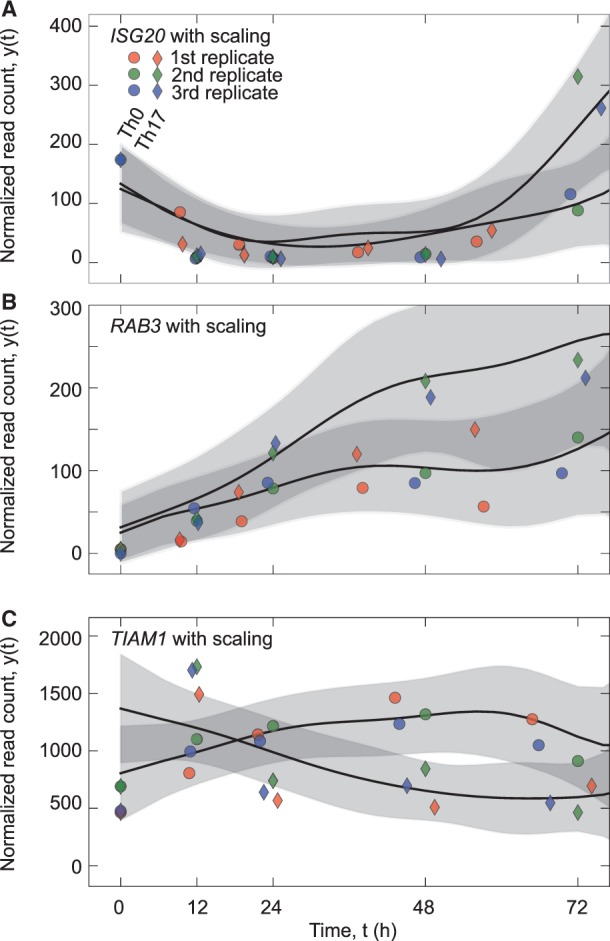
Examples of differentially expressed genes detected exclusively by DyNB**.** (**A**) The estimated smooth representation of *ISG20* dynamics with the time scaling. (**B** and **C**) Same as (A), but the depicted results are for the *RAB13* and *TIAM1* genes

Three representative examples detected by DyNB, but not identified by DESeq from timepoint-wise analysis with the aforementioned criteria, are shown in Figure 7. These genes illustrate the benefits of the time-scaling parameter. The gene *ISG20* has similar behavior as the *IL17A* gene, i.e. it is induced between the last two timepoints (48 and 72 h) but the activation is delayed in the first replicate. *ISG20* has been reported to have a role in Th17 cells (Pan *et al.*, 2013). The members of the RAB protein family, e.g. RAB3, are known to play a major role in protein-mediated transport and in fusion of intracellular structures and are highly expressed in various cells of immune system, especially after activation (Pei *et al.*, 2012). TIAM1 (T lymphoma invasion and metastasis protein 1) has shown to have a role in T-cell trafficking through Rac activation (Gérard *et al.*, 2009). On the contrary, Supplementary Figure S4 shows two representative genes, *KIF11* and *MAP1B*, which are detected by the timepoint-wise analysis, but not by the temporal analysis implemented in DyNB. Temporal analysis together with the possibility to account for variable differentiation efficiencies can filter out those genes for which the replicated Th0 and Th17 profiles are seemingly similar and thus likely false positives.

## 4 DISCUSSION AND CONCLUSIONS

We presented the first statistical method, DyNB, to study RNA-seq dynamics together with a method to correct for, or detect, different time scales between RNA-seq time-series datasets. DyNB is compared with a commonly used method, DESeq that relies on the same statistical assumptions but analyzes data from each timepoints separately and, therefore, ignores correlations between timepoints. As expected, the comparison showed that the agreement between the methods is high but at the same time temporal modeling approach has some benefits. The most notable advantage is the possibility to take into account different differentiation efficiencies between biological replicates. Indeed, many experimental systems in cell development and differentiation display subtle kinetic differences between replicates, which are not necessarily apparent until large-scale transcriptomics data are obtained. This method might critically help improve the interpretation of such experiments. Concerning future improvements, the proposed straightforward MCMC sampling scheme might lead to inefficient sampling if more parameters are marginalized. In those cases, sampling could be improved by using more elegant samplers, such as elliptical sampling (Murray *et al.*, 2009).

Our results show that a temporal analysis can bring insights into analysis of differentiation processes and help in the analysis of time-series datasets. We demonstrated applicability of DyNB by applying it to time series RNA-seq data from Th17 and Th0 lineages and identified novel Th17-specific genes. We used qRT-PCR to validate our computational predictions of sample-specific time scales. For example, by taking into account differences in differentiation efficiencies, we can identify a more complete set of differentially expressed genes. In turn, this improves our ability to discern subtle changes in regulatory pathways and broaden the scope of targets available for intervention.
